# Sex-specific effects of maternal metformin intervention during glucose-intolerant obese pregnancy on body composition and metabolic health in aged mouse offspring

**DOI:** 10.1007/s00125-022-05789-0

**Published:** 2022-09-16

**Authors:** Josca M. Schoonejans, Heather L. Blackmore, Thomas J. Ashmore, Lucas C. Pantaleão, Luciana Pellegrini Pisani, Laura Dearden, John A. Tadross, Catherine E. Aiken, Denise S. Fernandez-Twinn, Susan E. Ozanne

**Affiliations:** 1 Wellcome-MRC Institute of Metabolic Science-Metabolic Research Laboratories and MRC Metabolic Diseases Unit, University of Cambridge, Cambridge, UK; 2 Present Address: Department of Women and Children’s Health, King’s College London, London, UK; 3 Department of Bioscience, Laboratory of Nutrition and Endocrine Physiology, Federal University of São Paulo, Santos, Brazil; 4 Department of Pathology, University of Cambridge, Cambridge, UK; 5 Department of Obstetrics and Gynaecology, University of Cambridge, Cambridge, UK

**Keywords:** Developmental programming, Fatty liver, Gestational diabetes, Inflammation, Maternal obesity, Metformin, White adipose tissue

## Abstract

**Aims/hypothesis:**

Metformin is increasingly used to treat gestational diabetes (GDM) and pregnancies complicated by pregestational type 2 diabetes or polycystic ovary syndrome but data regarding long-term offspring outcome are lacking in both human studies and animal models. Using a mouse model, this study investigated the effects of maternal metformin intervention during obese glucose-intolerant pregnancy on adiposity, hepatic steatosis and markers of metabolic health of male and female offspring up to the age of 12 months.

**Methods:**

C57BL/6J female mice were weaned onto either a control diet (Con) or, to induce pre-conception obesity, an obesogenic diet (Ob). The respective diets were maintained throughout pregnancy and lactation. These obese dams were then randomised to the untreated group or to receive 300 mg/kg oral metformin hydrochloride treatment (Ob-Met) daily during pregnancy. In male and female offspring, body weights and body composition were measured from 1 month until 12 months of age, when serum and tissues were collected for investigation of adipocyte cellularity (histology), adipose tissue inflammation (histology and quantitative RT-PCR), and hepatic steatosis and fibrosis (histochemistry and modified Folch assay).

**Results:**

At 12 months of age, male Ob and Ob-Met offspring showed increased adiposity, adipocyte hypertrophy, elevated expression of proinflammatory genes, hyperleptinaemia and hepatic lipid accumulation compared with Con offspring. Male Ob-Met offspring failed to show hyperplasia between 8 weeks and 12 months, indicative of restricted adipose tissue expansion, resulting in increased immune cell infiltration and ectopic lipid deposition. Female Ob offspring were relatively protected from these phenotypes but Ob-Met female offspring showed increased adiposity, adipose tissue inflammation, hepatic lipid accumulation, hyperleptinaemia and hyperinsulinaemia compared with Con female offspring.

**Conclusions/interpretation:**

Maternal metformin treatment of obese dams increased offspring metabolic risk factors in a sex- and age-dependent manner. These observations highlight the importance of following up offspring of both sexes beyond early adulthood after interventions during pregnancy. Our findings illustrate the complexity of balancing short-term benefits to mother and child vs any potential long-term metabolic effects on the offspring when prescribing therapeutic agents that cross the placenta.

**Graphical abstract:**

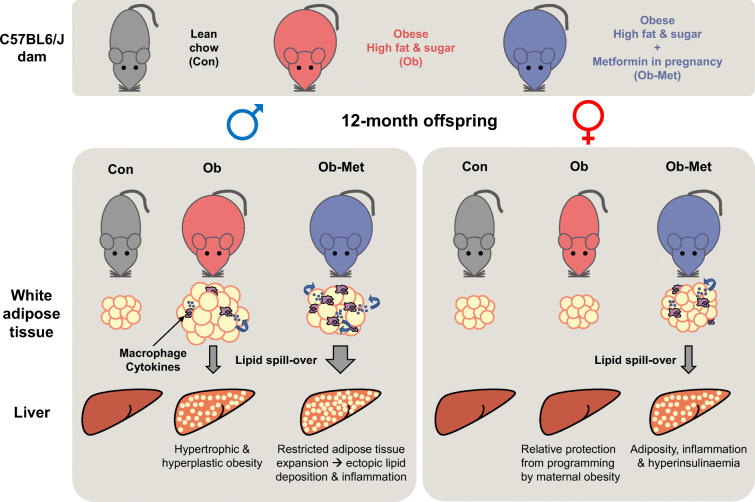

**Supplementary Information:**

The online version contains peer-reviewed but unedited supplementary material available at 10.1007/s00125-022-05789-0.

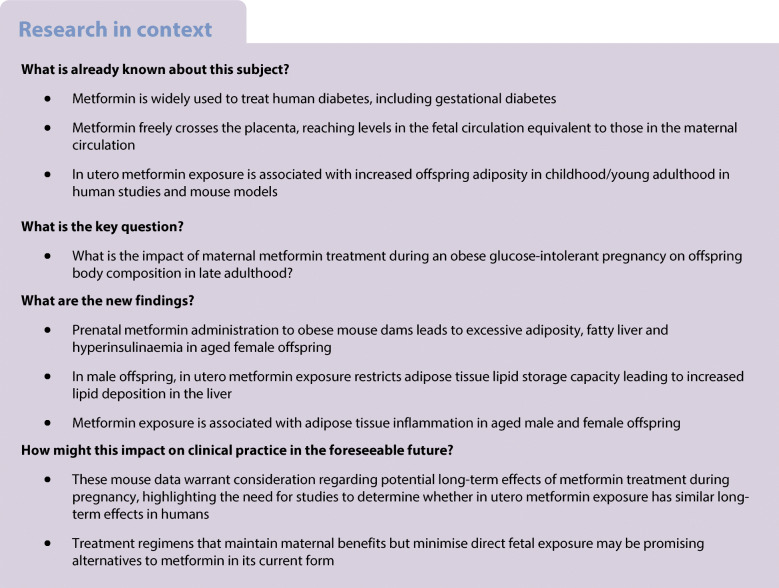



## Introduction

Gestational diabetes mellitus (GDM) is becoming more common, affecting over one in seven pregnancies worldwide [1]. GDM has immediate detrimental consequences for mother and neonate but is also increasingly recognised to have long-term adverse effects on exposed offspring [2]. Metformin is used as a pharmacological alternative to insulin treatment in GDM in many countries [3], with the benefits of being more affordable and easier to administer than insulin. Moreover, metformin may be continued during pregnancies complicated by type 2 diabetes and polycystic ovary syndrome (PCOS) [4]. Meta-analyses of RCTs comparing metformin with insulin or other treatments have found that metformin is safe for use in GDM with respect to pregnancy and early neonatal outcomes and that metformin has many immediate benefits including decreasing gestational weight gain, preventing pre-eclampsia, and decreasing incidence of macrosomia in newborns [4, 5]. However, metformin is readily transported across the placenta, so fetuses are rapidly exposed to concentrations similar to those in the maternal circulation [6], with limited knowledge of long-term consequences. A meta-analysis using data from RCTs comparing metformin with insulin in GDM showed that metformin decreases birthweight but leads to accelerated postnatal growth, ultimately resulting in increased childhood adiposity, warranting concerns into long-term metabolic consequences for offspring [5]. Lower mean birthweight and increased prevalence of small for gestational age babies were also found following gestational metformin treatment of women with type 2 diabetes, consistent with growth restriction in metformin-exposed pregnancy [7]. However, longer-term human follow-up is currently lacking: the oldest offspring of metformin-treated mothers with GDM or PCOS from which data are available are 9 or 5–10 years old, respectively [8, 9]. Model systems are therefore vital tools, and animal models with shorter lifespans are especially suited for investigating long-term outcomes of prenatal metformin treatment in offspring.

We have studied the effects of maternal metformin treatment during high-fat high-sugar diet-induced glucose-intolerant pregnancy [10, 11]. By ceasing metformin treatment immediately before delivery, this mimics the clinical management of GDM-complicated pregnancies in which metformin is discontinued after delivery when maternal hyperglycaemia generally resolves. Using this mouse model, we demonstrated that metformin transporters are expressed in both placental and fetal tissues and that fetuses are exposed to similar concentrations of metformin as their dams [11]. Metformin did not correct the growth restriction observed in offspring of obese dams despite improving maternal glucose tolerance, body composition and uterine blood flow as well as increasing gestation time [10, 11].

Several studies have previously investigated metformin interventions during rodent pregnancy (reviewed in [12]). Although these studies vary in design and findings, their results indicate that metformin has the potential to affect offspring body composition and metabolic health [12]. However, few studies looked at outcomes in aged offspring, and only in the context of lean pregnancy without diabetes [13]. No previous rodent studies have investigated offspring from obese metformin-treated pregnancies beyond the age of 5–7 months [14, 15]. In the latter studies [14, 15], metabolic phenotypes largely emerged during the second half of the study period, stressing the importance of longer follow-up; however, the effect of ageing in these studies was confounded by the introduction of a high-fat diet challenge in early adulthood. In our previous study, 8-week-old male offspring showed increased hyperplastic adiposity with adipose tissue inflammation, which was not observed in female offspring [10].

This study therefore aimed to investigate the effect of maternal metformin intervention during obese glucose-intolerant pregnancy on the body composition and metabolic health of male and female offspring until 12 months of age, using a mouse model of diet-induced obesity with impaired glucose tolerance during pregnancy.

## Methods

### Animal model

**Fig. 1:**
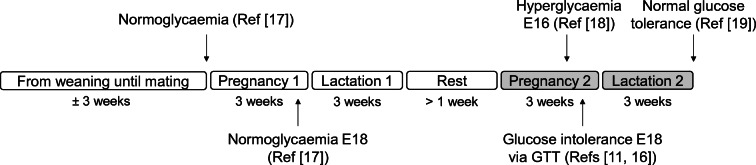
Schematic timeline representing the development of glucose intolerance in our mouse model of diet-induced obese pregnancy. Boxes refer to periods when mice were fed the high-fat high-sugar diet. The experimental pregnancy used for this study is Pregnancy 2. Reference numbers indicate the publications corresponding to the data. E16/18, embryonic day 16/18

All mouse work was performed according to the Home Office Animals (Scientific Procedures) Act 1986 Amendment Regulations 2012, after ethical review by the University of Cambridge Animal and Welfare Ethical Review Board. All mice were housed in individually ventilated cages in a temperature- and humidity-controlled room under a normal 12 h light–dark cycle. Using a previously described mouse model of maternal diet-induced obesity [16], specific pathogen-free C57BL/6J female mice (Charles River Laboratories, UK; RRID:IMSR_JAX:000664, bred in-house) were randomly weaned (by an animal technician who had no involvement in the data analysis) onto either a standard laboratory chow (RM1; 7% sugars, 3% fat) or an obesogenic diet composed of high-fat diet pellets (10% sugars, 20% fat), both from Special Dietary Services (UK), and sweetened condensed milk (55% sugar, 8% fat; Nestle, UK) supplemented with a vitamin and mineral pre-mix (AIN-93G-MX; Special Diets Services). Randomisation involved a technician (who was blind to the study outcomes) randomly weaning half of the females from each litter onto the control diet and the other half onto the obesogenic diet. Both groups were mated for a primary pregnancy at around 6 weeks of age, at which point dams are normoglycaemic [17]. Dams remain normoglycaemic during this first pregnancy [16]. Dams were mated for the experimental second pregnancy at least 1 week after weaning of their first litter and when they exceeded (obese, >12 g) or remained below (control, <5 g) critical thresholds of fat mass measured using time-domain NMR (TD-NMR; Bruker Minispec LF series; Bruker Optik, Germany). Dams were fed their respective control or obesogenic diets ad libitum throughout the experimental pregnancy and lactation. Previous work shows that these obese dams have increased adiposity at mating [10, 11, 16] and become hyperglycaemic [18] and glucose-intolerant [11, 16] in their second pregnancy. Glucose intolerance resolves by the end of lactation [19] (see Fig. 1 for schematic timeline of glucose homeostasis). As described [10], dams fed the obesogenic diet were randomised by a technician (who was blind to the study outcomes) to the untreated group or to daily treatment with 300 mg/kg metformin hydrochloride orally (MP Biomedicals, USA) supplemented in the condensed milk (adjusted twice weekly based on intake). This dose is comparable to 1700 mg for a 70 kg human [20] and results in serum metformin concentrations within the clinical range observed in human pregnancy [11, 21]. Metformin treatment was provided 1 week pre-mating until embryonic day 18.5, 1 day before normal-term delivery. Data regarding dams that generated offspring for the current study were published previously [10]. Detailed characterisation of the maternal metformin intervention model (including improvement of maternal glucose intolerance with metformin), in a separate cohort of animals generated using an identical experimental design, has recently been published [11]. Litter size was standardised by culling to six pups per litter on postnatal day 2. Male and female offspring were weaned onto RM1 diet at 3 weeks of age and housed in same-sex littermate pairs where possible. Mice were weighed monthly; the mean littermate body weight is reported. Body composition of one sibling was assessed monthly using TD-NMR until 6 months of age. Diet intake was measured between 11 and 11.5 months. At 12 months of age (mean±SEM 367.1±0.4 days, range 351–378), in the morning one sibling was euthanised via exposure to CO_2_ gas in a rising concentration following a 16 h fast in a clean cage, while the other sibling was euthanised by cervical dislocation in the fed state. Tissues were weighed and formalin-fixed (fed siblings) or snap-frozen (fasted siblings) on dry ice.

### mRNA levels

RNA extraction from gonadal white adipose tissue (gWAT), cDNA synthesis (from 450 ng RNA) and quantitative RT-PCR (RT-qPCR, using cDNA diluted 1:40 in nuclease-free water) were performed as described [10]. For analysis of RT-qPCR results, the comparative C_t_ method was used normalised against expression of *Ppia*, which did not differ between groups. Data are presented as expression relative to the relevant control group. Primer sequences are shown in Table 1, either obtained from the literature (*Itgax* [also known as *Cd11c*], *Adgre1* [also known as *F4/80*], *Tnf* [22] and *Ppia* [23]) or designed using Primer-BLAST software [24] (*Ccl2*; also known as *Mcp1*).

**Table 1:** Primer sequences used for real-time RT-qPCR

Gene	Forward 5′-3′	Reverse 5′-3′	Origin
*Adgre1*	CACTTCCAAGATGGGTTAACATCC	CTGCCATCAACTCATGATACCCT	Alfaradhi et al [22]
*Ccl2*	CAGATGCAGTTAACGCCCCA	TGAGCTTGGTGACAAAAACTACAG	This paper
*Itgax*	TGCTGTTGGGGTTTGTTTCTTG	CGAACTCAGCACCGTCCAT	Alfaradhi et al [22]
*Ppia*	GTCCAGGAATGGCAAGACCA	GGGTAAATGCCCGCAAGTC	Mennitti et al [23]
*Tnf*	AAGTTCCCAAATGGCCTCCC	CACTTGGTGGTTTGCTACGA	Alfaradhi et al [22]

Primer pair for *Ccl2* was generated for this paper using Primer-BLAST software [24]

### Histological analysis

Offspring gWAT and livers were formalin-fixed, processed, embedded and sectioned. H&E-stained gWAT sections (3 μm) were scanned using an Axioscan digital slide scanner (Zeiss, Germany); whole sections were examined for the presence of crown-like structures (CLS) and cells were sized using HALO software (Indica Labs, USA) as described [10]. Estimated adipocyte number and the percentage of adipocytes surrounded by CLS were calculated based on published methods [10]. H&E-stained liver sections (5μm) were analysed for lipid droplet content using HALO software and scored for steatosis by a pathologist blinded to experimental group and offspring sex, leading to classification into four grades: grade 0 (absent, <5%); grade 1 (mild, <30%); grade 2 (moderate, <60%); or grade 3 (severe, >60% lipid within hepatocytes) [25]. Similarly, Picrosirius Red-stained liver sections (5 μm) were analysed for hepatic collagen content using QuPath v.0.2.3 (University of Edinburgh, UK [26]) and sections were scored for fibrosis, leading to classification into four categories: absent; mild (only centrilobular/pericellular fibrosis); moderate (centrilobular and portal fibrosis); or severe (bridging fibrosis) [27].

### Folch assay

Lipid content was measured using the modified Folch method as described [28].

### Serum analysis

Cardiac puncture was performed to collect fasted terminal blood, and serum was collected after centrifugation (3000 *g*, 2×3 min). Serum insulin and leptin were measured using an Ultra-Sensitive Mouse Insulin ELISA Kit and Mouse Leptin ELISA Kit (Crystal Chem, USA). Serum cholesterol and triglycerides were measured by the Core Biochemical Assay Laboratory (University of Cambridge).

### Statistical analysis

Data are presented as mean ± SEM and analysed using Prism v.9.0 software (GraphPad, USA) using one-way ANOVA, two-way ANOVA or non-parametric alternatives where appropriate. Correlation was assessed using two-tailed Spearman correlation in Prism v9.0. Hepatic histology results were analysed using multinomial regression analysis with absent steatosis as the referent level and offspring sex and maternal diet as model co-variates. A *p* value of <0.05 was considered statistically significant, with trends included in figures if *p*<0.1. In all cases, *n* refers to the number of independent litters represented, meaning one dam only contributed one male and/or female for use in a particular experiment. One mouse from the male Ob-Met group had to be culled prior to the study endpoint and was therefore excluded from analysis. The total number of mice used for this study is *n*=54 dams (*n*=18 control, *n*=21 obese, *n*=15 obese metformin-treated dams) providing *n*=67 male offspring and *n*=71 female offspring. Outliers were determined per outcome by the Grubb’s or ROUT method and excluded from analysis. Numbers and outliers for each specific outcome measure are included within the results and/or figure legends.

## Results

### Maternal obesity and metformin exposure lead to adiposity in offspring

**Fig. 2:**
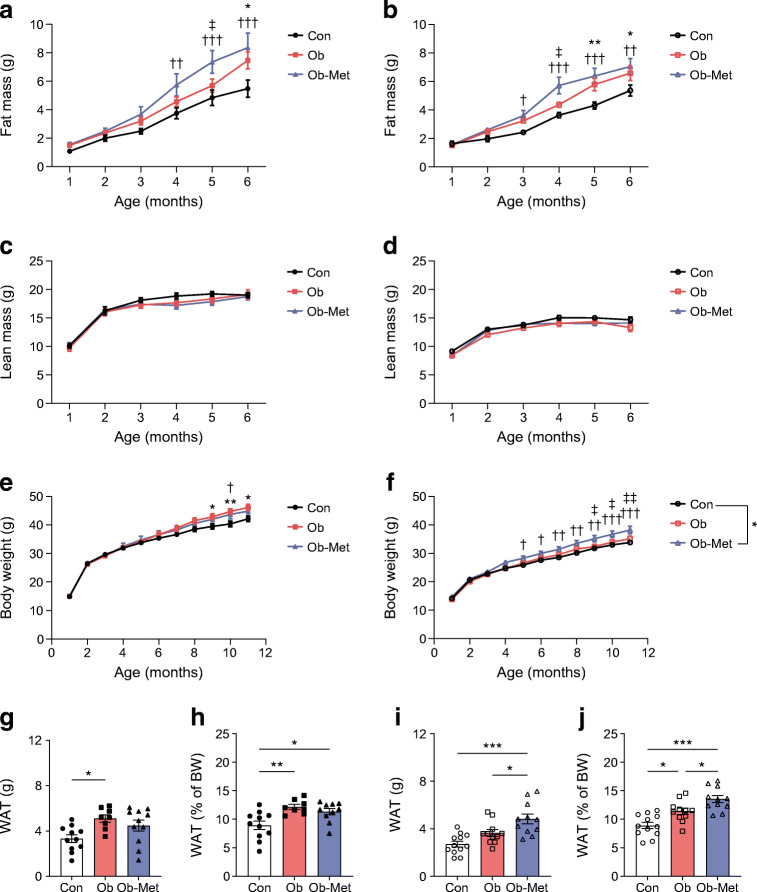
Mouse offspring body composition. (**a**–**d**) Fat mass (**a**, **b**) and lean mass (**c**, **d**) of male (**a**, **c**) and female (**b**, **d**) offspring until 6 months of age by TD-NMR. Numbers are *n*=9–12 (male Con), *n*=9–12 (male Ob), *n*=9–11 (male Ob-Met), *n*=11 or 12 (female Con), *n*=10–12 (female Ob) and *n*=11 or 12 (female Ob-Met) independent litters per group. (**e**, **f**) Body weight of male (**e**) and female (**f**) offspring until 12 months of age (*n*=12 independent litters per group; data are littermate means). *p<0.05 and **p<0.01, Con vs Ob; ^†^*p*<0.05, ^††^*p*<0.01 and ^†††^*p*<0.001, Con vs Ob-Met; ^‡^*p*<0.05 and ^‡‡^*p*<0.01 Ob vs Ob-Met (two-way ANOVA with Tukey’s multiple comparison test). (**g**–**j**) total weight of WAT depots collected at 12 months of age in male (**g**, **h**) and female (**i**, **j**) offspring in absolute terms and relative to offspring body weight (*n*=10–12 independent litters per group except for *n*=8 for male Ob group). An outlier was excluded from (**g**) male Ob-Met (ROUT method, outlier excluded value 4.71 g). *p<0.05, **p<0.01 and ***p<0.001 (one-way ANOVA with Tukey’s multiple comparison test). Black circles, Con (offspring of control-fed dams); pink squares, Ob (offspring of obese dams); blue triangles, Ob-Met (offspring of obese metformin-treated dams); closed symbols, male offspring; open symbols, female offspring. BW, body weight

Male and female offspring of obese dams showed increased fat mass from 6 months and 5 months of age, respectively (Fig. 2a,b). Metformin intervention led to earlier appearance of this adiposity, introducing increased fat mass compared with Con from 4 and 3 months of age in male and female offspring, respectively (Fig. 2a,b). There were no differences in lean mass or body weight early in life in either sex (Fig. 2c–f). However, increased body weight was noted from 9 months onwards in male Ob offspring and from 10 months onwards in male Ob-Met offspring (Fig. 2e). Female Ob-Met offspring diverged in body weight from other groups at a younger age: from 5 and 9 months of age compared with Con and Ob offspring, respectively (Fig. 2f). There was no difference in body weight between female Ob and Con offspring at any age. Food intake was not measurably different between groups (see electronic supplementary material [ESM] Table 1).

At 12 months of age, the combined absolute weight of white adipose tissue (WAT) depots (gonadal, intraperitoneal, retroperitoneal and inguinal subcutaneous) was increased by maternal obesity in male offspring (Fig. 2g) and by both maternal obesity and metformin treatment in female offspring (Fig. 2i) when compared with Con offspring. Combined WAT weights were significantly increased both by maternal obesity and metformin treatment in male and female offspring when compared with Con offspring (Fig. 2h,j). In female Ob-Met offspring adiposity was also increased compared with Ob offspring (Fig. 2i,j). These phenotypes were consistent across different WAT depots and brown adipose tissue (BAT, ESM Table 2) suggesting a global adiposity phenotype.

### Maternal metformin treatment leads to gWAT inflammation in male and female 12-month-old offspring

**Fig. 3:**
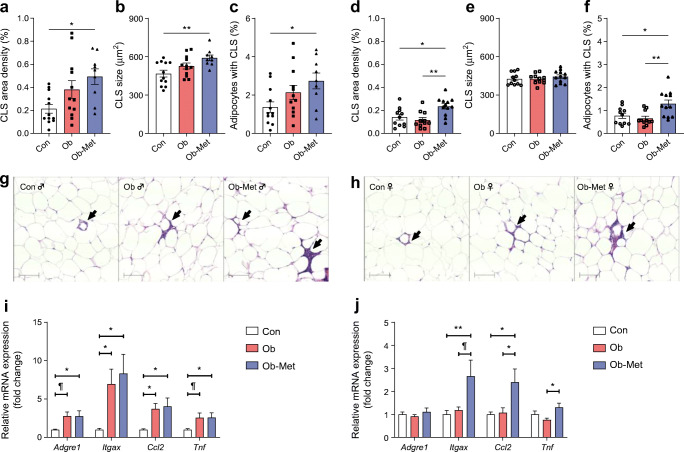
Immune cell infiltration and inflammation in 12-month-old mouse gonadal adipose tissue. (**a**–**h**) Immune cell infiltration into gWAT of 12-month-old male (**a**–**c**) and female (**d**–**f**) offspring as assessed by the presence of CLS as seen on the representative images for male (**g**) and female (**h**) offspring (*n*=9–12 independent litters per group). Scale bar, 100 μm. Area density of CLS in WAT (**a**, **d**), CLS size (**b**, **e**) and percentage of adipocytes surrounded by CLS (**c**, **f**) are shown. (**i**, **j**) mRNA expression of macrophage markers and proinflammatory genes in gonadal WAT of 12-month-old male (**i**) and female (**j**) offspring relative to the expression of *Ppia* (fold change using the comparative C_t_ method) and expressed relative to Con offspring (*n*=11 or 12 independent litters except for *n*=8 male Ob offspring). Outliers were excluded as follows: from (**i**) *Adgre1*, *Cd1c* and *Ccl2* for male Con (Grubb’s method, outlier excluded values 2.178-, 7.088- and 2.391-fold, respectively); from (**j**) *Ccl2* and *Tnf* for female Ob (Grubb’s method, outlier excluded values 4.790- and 2.440-fold, respectively); and from (**j**) *Itgax* for one female Con and one female Ob (Grubb’s method, outlier excluded values 4.059- and 3.671-fold, respectively). *p<0.05 and **p<0.01; ^¶^0.05<*p*<0.10 (one-way ANOVA with Tukey’s multiple comparison test). Circles, Con (offspring of control-fed dams); squares, Ob (offspring of obese dams); triangles, Ob-Met (offspring of obese metformin-treated dams); closed symbols, male offspring; open symbols, female offspring

The total area density of CLS was increased in male Ob-Met offspring, indicating macrophage infiltration into gWAT (Fig. 3a). This was related to increased CLS size as well as an increase in the number of adipocytes surrounded by CLS (Fig. 3b,c). Expression of *Adgre1* (a macrophage marker), *Itgax* (an M1-type marker), *Ccl2* (a chemokine) and *Tnf* (a cytokine) was also increased in male Ob-Met offspring (Fig. 3i). Male Ob offspring did not show histological evidence of enhanced macrophage infiltration (Fig. 3a–c) compared with Con offspring despite upregulated expression of *Itgax* and *Ccl2* (Fig. 3i).

In female Ob-Met offspring, immune cell infiltration in gWAT was upregulated compared with both Con and Ob offspring (Fig. 3d). CLS size was unaffected (Fig. 3e) but the percentage of adipocytes surrounded by CLS, a marker of adipocyte death [29], was increased in female Ob-Met offspring (Fig. 3f). This was accompanied by increased expression of *Itgax*, *Ccl2* and *Tnf* in gWAT (Fig. 3j).

### Maternal obesity and metformin affect gWAT cellularity in a sex-specific manner

**Fig. 4:**
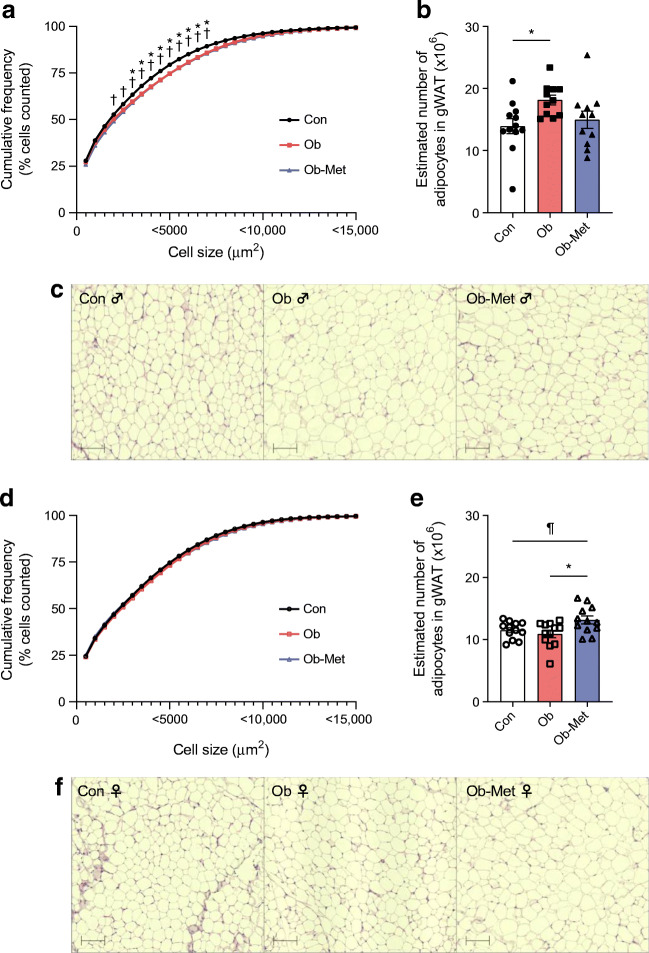
Cellularity of 12-month-old mouse offspring gWAT. (**a**, **d**) Cumulative frequency distribution of gonadal adipocyte size in male (**a**) and female (**d**) offspring shown as a curve reflecting the percentage of cells that falls below a particular adipocyte size, with representative images of H&E-stained sections for male (**c**) and female (**f**) offspring (*n*=11 or 12 independent litters per group). Scale bar, 100 μm. *p<0.05 for Con vs Ob; ^†^*p*<0.05 for Con vs Ob-Met; ^¶^0.05<*p*<0.10 (two-way ANOVA with Tukey’s multiple comparison test). (**b**, **e**) Estimated adipocyte number in the collected gWAT depot for male (**b**) and female (**e**) offspring. An outlier was excluded in (**b**) from male Ob-Met (Grubb’s method, outlier excluded value 3.27×10^6^ cells). *p<0.05 (one-way ANOVA with Tukey’s multiple comparison test). Black circles, Con (offspring of control-fed dams); pink squares, Ob (offspring of obese dams); blue triangles, Ob-Met (offspring of obese metformin-treated dams); closed symbols, male offspring; open symbols, female offspring

Adipocyte hypertrophy was observed in both Ob and Ob-Met male 12-month-old offspring, demonstrated by a rightward shift in the cumulative frequency distribution favouring larger adipocytes in gWAT (two-way ANOVA; Fig. 4a). Moreover, estimated number of adipocytes was increased in male Ob but not Ob-Met offspring compared with Con offspring (Fig. 4b). When comparing this to adipocyte numbers from 8-week-old mice published previously [10], there was a significant effect of age, maternal environment and an interaction between male offspring age and the maternal environment (ESM Table 3). Post hoc analysis revealed that age-related hyperplasia had taken place in Con and Ob mice; this was absent in male Ob-Met offspring, indicating that they had exhausted their gWAT hyperplastic expansion capacity early in adult life.

There were no differences in adipocyte size when comparing Con, Ob and Ob-Met female offspring (Fig. 4d). Estimated adipocyte number was increased in female Ob-Met offspring (Fig. 4e). When comparing this with female adipocyte numbers from 8-week-old mice [10], there was a significant effect of age and maternal environment (ESM Table 3). As there was no difference in adipocyte number at 8 weeks, this suggests hyperplastic expansion of the female Ob-Met gWAT depot after this age.

### Hepatic lipid accumulation is increased in Ob-Met offspring of both sexes

**Fig. 5:**
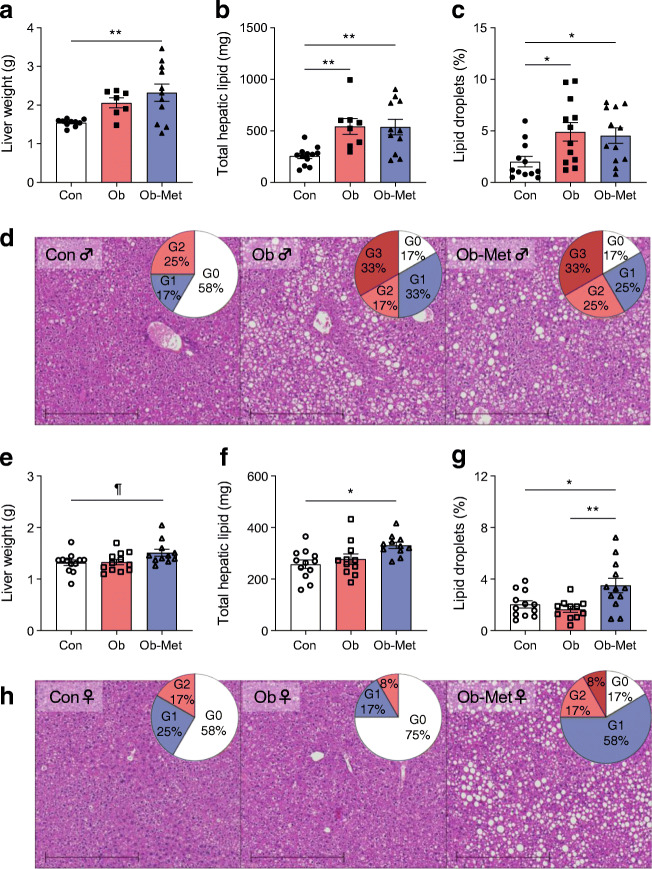
Hepatic lipid accumulation in 12-month-old mouse offspring. (**a**, **b**, **e**, **f**) Fasted liver weight (**a**, **e**) and corresponding total hepatic lipid content as assessed by Folch assay (**b**, **f**) in male (**a**, **b**) and female (**e**, **f**) offspring (*n*=11 or 12 independent litters per group except for *n*=7 or 8 male Ob offspring). (**c**, **d**, **g**, **h**) Area density of lipid droplets in H&E-stained liver tissue (**c**, **g**) with representative images (**d**, **h**) of male (**c**, **d**) and female (**g**, **h**) offspring (*n*=11 or 12 independent litters per group). Scale bar, 500 μm. Pie charts show percentages of pathological scoring for hepatic steatosis: white, G0 (grade 0, absent); blue, G1 (grade 1, mild); light red, G2 (grade 2, moderate); dark red, G3 (grade 3, severe). Outliers were excluded from (**a**) for male Con offspring (ROUT method, 2.04 g and 1.08 g) and male Ob offspring (ROUT method, 3.9 g). An outlier was excluded from (**g**) for female Ob offspring (ROUT method, outlier excluded value 5.03%). *p<0.05 or **p<0.01; ^¶^0.05<*p*<0.10 (one-way ANOVA with Tukey’s multiple comparison test). Closed symbols, male offspring; open symbols, female offspring

Liver weight was increased in male (Fig. 5a) and female (Fig. 5e) Ob-Met offspring, although this did not reach statistical significance for female offspring. Total hepatic lipid content was increased in livers from both Ob and Ob-Met male offspring compared with Con offspring, and this was confirmed by histological analysis of lipid droplets (Fig. 5b–d). In female offspring, hepatic lipid accumulation was only observed in Ob-Met offspring (Fig. 5f–h). Total hepatic lipid content significantly correlated to indices of body composition, serum hormones and gWAT inflammation in male offspring, and to body weight, visceral WAT and leptin (but not serum hormones or proinflammatory gene expression) in female offspring (Table 2). Compared with female offspring, there was increased incidence of moderate/severe steatosis in male offspring (OR 4.99 [95% CI 1.39, 17.96], *p*<0.05). There was no difference in steatosis in Ob compared with Con offspring (both sexes combined) but there was increased incidence of both mild (OR 7.24 [95% CI 1.53, 34.30], *p*<0.05) and moderate/severe steatosis (OR 8.21 [95% CI 1.60, 42.06], *p*<0.05) in Ob-Met offspring (both sexes combined). Collagen content and hepatic fibrosis as assessed by Picrosirius Red staining was not different between groups (ESM Fig 1).

**Table 2:** Correlation between total hepatic lipid accumulation and metabolic/inflammatory variables in 12-month-old mouse offspring

Variable	Male offspring			Female offspring		
	*r*	*p*	*n*	*r*	*p*	*n*
BW (g)	0.8982	<0.001	31	0.6605	<0.001	34
VAT (g)	0.9101	<0.001	30	0.6726	<0.001	34
Insulin (pmol/l)	0.6957	<0.001	28	0.3376	0.0633	31
Leptin (ng/ml)	0.9212	<0.001	30	0.6305	<0.001	33
*Adgre1*	0.9018	<0.001	30	0.2581	0.1405	34
*Ccl2*	0.8549	<0.001	30	0.4835	0.0044	33
*Itgax*	0.9159	<0.001	30	0.4255	0.0152	32
*Tnf*	0.8827	<0.001	31	0.342	0.0514	33

Correlation was assessed using two-tailed Spearman correlation between total hepatic lipid content (measured using Folch assay) with the following variables: body weight at post mortem, weight of collected VAT (sum of gonadal, intraperitoneal and retroperitoneal depots), serum insulin levels, serum leptin levels and expression of *Adgre1*, *Itgax*, *Ccl2* and *Tnf* in gonadal adipose tissue relative to Con offspring

BW, body weight; VAT, visceral adipose tissue

### Maternal metformin treatment increases insulin levels in female but not male offspring

There were no differences in serum lipids or insulin in 12-month-old male offspring (Table 3). Serum leptin was increased in both Ob and Ob-Met male offspring compared with Con offspring, consistent with their increased adiposity. In contrast to male offspring, 12-month-old female Ob-Met offspring displayed significantly increased serum insulin compared with female Con offspring (Table 3). Serum leptin was also increased in female Ob-Met but not Ob offspring.

**Table 3:** Serum analysis

Variable	Male offspring				Female offspring			
	Con (*n*=*11 or 12)*	Ob (*n*=6–8)	Ob-Met (*n*=9–11)	*p* value (ANOVA)	Con (*n*=10–12)	Ob (*n*=11 or 12)	Ob-Met (*n*=10 or 11)	*p* value (ANOVA)
Glucose (mmol/l)^a^	6.9±0.3	7.6±0.4	6.8±0.2	NS	6.6±0.2	6.5±0.2	6.5±0.2	NS
Insulin (pmol/l)^b^	146±14	198±28	217±41	NS	101±16	134±17	180±23*	0.0227
Cholesterol (mmol/l)^b,c^	3.1±0.1	3.4±0.2	3.6±0.3	NS	2.1±0.1	2.0±0.1	2.0±0.1	NS
Triglycerides (mmol/l)^b^	1.09±0.06	0.99±0.07	1.09±0.06	NS	0.97±0.05	1.06±0.06	1.12±0.05	NS
NEFA (mmol/l)	1.60±0.06	1.64±0.12	1.61±0.07	NS	1.35±0.05	1.28±0.05	1.31±0.05	NS
Leptin (ng/ml)^d^	16.6±2.8	34.8±4.5*	33.7±5.5*	<0.001	23.7±2.5	27.9±3.2	49.3±5.8***^,†^	<0.001

All data reflect serum from 16 h fasted mice at 12 months of age and are represented as mean ± SEM

Outliers were excluded (Grubb’s method) as follows: from male Con for insulin (396 pmol/l); from male Ob for cholesterol (5.5 mmol/l); from female Con for glucose (excluded value 9.7 mmol/l), insulin (337 pmol/l) and NEFA (2.33 mmol/l); from female Ob for NEFA (2.22 mmol/l); and from female Ob-Met for glucose (3.0 mmol/l) and for NEFA (2.32 mmol/l)

^a^Serum was not collected for one male Ob mouse therefore *n*=8 for glucose compared with *n*=7 for other variables

^b^One sample from a male Ob-Met mouse was not analysed for cholesterol, triglycerides or insulin

^c^Cholesterol was not detectable for one male control mouse and female control mouse (<1.3 mmol/l) and therefore these data were excluded from analysis

^d^One sample from a female Ob mouse was not analysed for leptin

*p<0.05 and ***p<0.001 vs Con offspring; ^†^*p*<0.01 vs Ob offspring (one-way ANOVA with Tukey’s multiple comparison test)

## Discussion

Our study showed that maternal obesity and maternal metformin treatment during obese pregnancy caused long-term increases in offspring adiposity in an age- and sex-specific manner. Male Ob offspring showed increased adiposity, upregulated proinflammatory gene expression in gWAT, and hepatic lipid accumulation at 12 months of age. Adiposity in aged male Ob-Met offspring was associated with more severe adipocyte dysfunction (worse inflammation and early-life hyperplastic response), leading to hepatic steatosis and lipid accumulation that was correlated with adipose tissue and metabolic variables. While female Ob offspring did not show gWAT or liver dysfunction, female Ob-Met offspring had increased adiposity, gWAT inflammation and hyperplasia, hepatic steatosis, hyperinsulinaemia and hyperleptinaemia. Table 4 summarises these findings.

**Table 4:** Summary of findings in offspring at 12 months of age

Variable	Male offspring			Female offspring		
	Ob vs Con	Ob-Met vs Con	Ob-Met vs Ob	Con vs Ob	Con vs Ob-Met	Ob vs Ob-Met
Adipose tissue						
Adiposity	↑	↑	↔	↑	↑↑	↑
Hypertrophy	↑	↑	↔	↔	↔	↔
Hyperplasia	↑	↔	↔	↔	↑	↔
Inflammation	↑	↑↑	↔	↔	↑	↑
Liver						
Hepatomegaly	↔	↑	↔	↔	(↑)	↔
Lipid accumulation	↑	↑	↔	↔	↑	↑
Serum						
Insulin	↔	↔	↔	↔	↑	↔
Leptin	↑	↑	↔	↔	↑	↑

This table summarises the sex-specific findings presented in the current manuscript

↑, upregulated; ↑↑, further upregulated; ↔, unchanged; arrows within parentheses indicate changes that do not reach statistical significance (0.05<*p*<0.1)

Our work demonstrates clear sex differences in the programming of obesity and fatty liver by maternal obesity, while also highlighting the importance of age to the development of programmed phenotypes. The WAT expansion in Ob male offspring was associated with increased proinflammatory gene expression at 12 months (not present at 8 weeks [10]), in accordance with ageing promoting a proinflammatory environment [30]. In contrast, female Ob offspring were protected against the development of obesity, gWAT inflammation and hepatic abnormalities. Sexual dimorphism is often described in the context of developmental programming, with the male sex generally being more vulnerable to programmed effects [31]. This could be related to biologically underpinned differences, such as male mice growing more rapidly in utero, differential effects of sex steroids or male mice ageing faster than female mice [31].

Metformin is widely used in the developed world to treat GDM [3] and has proven beneficial for treatment of other pregnancy indications including PCOS and pre-eclampsia [9, 32, 33]. It is an attractive alternative to insulin in populations where access to insulin is limited or subject to financial barriers [34] and many women prefer metformin to insulin as it does not require injection [35]. Our experimental model simulates certain features of human GDM, including impaired glucose tolerance in pregnancy [11, 16] that is no longer present after weaning [19], and our dosing protocol leads to maternal circulating metformin concentrations similar to those in humans (with equilibration to the fetal circulation) [11]. This is consistent with detection of known metformin transporters in murine and human placenta [11, 36]. However, the initiation of metformin treatment around conception may more closely resemble treatment of pregnant women with type 2 diabetes or PCOS and our offspring data are therefore also relevant to metformin use in those clinical contexts. The difference in timing of WAT development between rodents and humans could be a limitation to clinical relevance. However, adipocyte lineage commitment largely occurs in gestation in both humans and rodents, and thus may be similarly influenced by intrauterine metformin [37]. Importantly, our data parallel outcomes of human trials. Individual RCTs for GDM and PCOS pregnancies have shown increased adiposity in young metformin-exposed offspring compared with insulin and placebo groups, respectively [8, 9, 38], and these findings have been confirmed by a meta-analysis of trials in GDM [5].

The maternal metformin intervention did not correct the adiposity observed in 12-month-old male offspring exposed to maternal obesity but induced a more inflammatory gWAT phenotype. This was reflected by presence of CLS and upregulation of M1 (*Itgax*, *Tnf*) and migratory markers (*Ccl2*) alongside the macrophage marker *Adgre1,* indicating recruitment of proinflammatory macrophages to hypertrophied WAT. After macrophage infiltration, inflammation can be propagated by dysfunctional adipocytes and activated macrophages [39], potentially explaining the most severe inflammation in Ob-Met male offspring. While Con and Ob male offspring showed significant increases in adipocyte number between 8 weeks and 12 months of age, male Ob-Met offspring had more adipocytes at 8 weeks but failed to elicit compensatory hyperplasia with ageing. This suggests their limit of WAT hyperplastic expansion capacity is reached by young adulthood (further supported by WAT depot weights being lower than in male Ob offspring at 12 months). The restricted WAT expandability resulted in ectopic lipid deposition in the livers of male Ob-Met offspring. This may result from direct effects of metformin on adipocyte progenitors in utero. Metformin decreases maturation and differentiation of mouse and human pre-adipocytes in vitro [40] and directly inhibits adipocyte lineage commitment [41]. Suppression of the number of adipocyte progenitors by metformin in utero could therefore explain the restricted WAT expandability, premature reliance on hyperplastic adipose tissue expansion, and hepatic lipid deposition in metformin-exposed male offspring. Increased mesenteric adiposity, liver weight and hepatic expression of lipogenic genes was previously reported in 20-week-old male and female offspring of metformin-treated chow-fed dams [20].

Female offspring exposed in utero to metformin exhibited an adiposity phenotype characterised by hyperinsulinaemia, hyperleptinaemia, gWAT inflammation and ectopic lipid deposition. The excessive WAT expansion in Ob-Met female offspring resulted from hyperplasia rather than hypertrophy. Sexual dimorphism in ageing dynamics might explain why, unlike the male offspring, female Ob-Met offspring retained the ability to elicit hyperplasia between 8 weeks and 12 months. This compensatory hyperplasia was insufficient to cope with expansion demands, as evidenced by the hepatic steatosis and increased proinflammatory signature of WAT in female Ob-Met offspring. The increase in CLS-surrounded adipocytes indicates increased adipocyte death in female Ob-Met offspring WAT [29]. The presence of hyperinsulinaemia in female Ob-Met offspring at 12 months of age is distinct from the previously reported phenotype at 8 weeks, where no difference was observed [10]. Since obesity and increased fat mass are strongly associated with these phenotypes [42], the abnormalities in insulin homeostasis in 12-month-old female Ob-Met offspring are likely secondary to the development of obesity. The increased adiposity in female Ob-Met offspring could result from alterations in energy expenditure, energy intake or nutrient assimilation in the intestines (alterations in BAT thermogenic capacity and intestinal microbiota have been reported following early-life metformin exposure [15, 43]). We found no detectable differences in food intake in 11-month-old offspring of either sex, and did not assess energy expenditure.

A striking finding of this study is that adiposity was more strongly induced by in utero metformin in aged female offspring and accompanied by hyperinsulinaemia in female but not male Ob-Met offspring. Most previous rodent studies investigating metformin interventions reveal similar outcomes in both sexes, although few studied offspring until 12 months of age. Since our study period coincides with the onset of oestropause [44], recent loss of oestrogen’s protective anti-inflammatory and pro-adipogenic effects on WAT [45] might have exacerbated the phenotype in female Ob-Met offspring, while intervention effects in male offspring may be masked by additional programming effects induced by maternal obesity. Metformin may also affect ageing dynamics in exposed offspring in a sex-specific manner, as suggested by a study in which repeated metformin injections were administered to neonatal mice [46]. We know of only one other study that described clear sex differences, with increased susceptibility in female offspring: prenatal metformin treatment to genetically obese neuropeptide Y-overexpressing dams caused adiposity and glucose intolerance in female but not male offspring at 7 months of age [15]. Others reported sex-specific timing of in utero metformin intervention: in lean glucose-tolerant pregnancy there were beneficial effects on insulin homeostasis in young adult metformin-exposed male offspring, and these effects weakened with age; in female offspring improvements in metabolic function only appeared at 15 months [13].

To our knowledge, our model is the most clinically relevant model of metformin intervention during maternal diet-induced glucose-intolerant pregnancy that is currently reported, with treatment dose and maternal serum concentrations comparable with those in human studies [11]. The main strength is the long-term follow-up of both male and female offspring, providing causal evidence for the development of obesity beyond young adulthood. This is especially pertinent as most phenotypes emerge after 6 months of age, a common maximum endpoint in developmental programming studies. This prolonged follow-up revealed age-sensitive sexually dimorphic effects of maternal obesity and metformin intervention. Although offspring were followed up until 12 months of age, the lifespan of mice is around 2 years and therefore this equates to middle age [47]. Since the age of offspring heavily influenced metabolic outcomes, it is important that future studies assess effects beyond 12 months.

Metformin treatment during a pregnancy complicated by GDM has clear short-term beneficial effects beyond glycaemic control, including decreased gestational weight gain (benefiting a subsequent pregnancy by preventing excessive interpregnancy weight gain [48]), lower incidence of pre-eclampsia, and improved neonatal outcomes [4, 49]. However, the findings of offspring adiposity and fatty liver resulting from maternal metformin exposure are concerning, as both childhood and adult obesity are an increasing problem worldwide [50]. Therefore, the relative short-term benefits and potential adverse long-term metabolic effects must be weighed against one another. It is vital that the outcomes investigated in the current study are addressed in human trials, as we cannot exclude species-specific differences, or that the intervention might have different effects depending on clinical indication or timing of metformin prescription. Offspring follow-up beyond childhood is therefore crucial in human clinical trials.

### Conclusions

Metformin exposure in utero during diet-induced obese pregnancy increased metabolic risk factors in a sex- and age-dependent manner. Our work highlights the importance of following up offspring of both sexes throughout the life course, in addition to immediate effects on mother and fetus, and illustrates the complexity of balancing short-term benefits of therapeutic agents that cross the placenta vs any long-term metabolic risks. Alternative treatment regimens or formulations that retain maternal benefits but limit fetal exposure to metformin might be promising areas of future research.

## Supplementary information

ESM (PDF 351 kb)

## Data Availability

The datasets generated during and/or analysed during the current study are available from the corresponding author on reasonable request.

## Abbreviations

BAT: Brown adipose tissue
CLS: Crown-like structures
GDM: Gestational diabetes mellitus
gWAT: Gonadal white adipose tissue
PCOS: Polycystic ovary syndrome
RT-qPCR: Quantitative RT-PCR
TD-NMR: Time-domain NMR
WAT: White adipose tissue
