# Supplementary material for: Sex-specific effects of maternal metformin intervention during glucose-intolerant obese pregnancy on body composition and metabolic health in aged mouse offspring
Source: Diabetologia. 2022 Sep 16;65(12):2132–45. doi: 10.1007/s00125-022-05789-0 (PMC9630251; doi:10.1007/s00125-022-05789-0)
Supplement: Supplementary file 1 — (PDF 351 kb) [file 125_2022_5789_MOESM1_ESM.pdf]

ELECTRONIC SUPPLEMENTARY MATERIAL (ESM)

ESM Table 1: Offspring energy intake

	Con	Ob	Ob-Met	*p*-value
**Males (kJ)**	785 [746-866] *n=12*	839 [801-914] *n=9*	849 [741-928] *n=12*	ns
**Females (kJ)**	690 [676-707] *n*=12	696 [659-751] *n=12*	709 [658-790] *n=11*	ns

Energy intake data was collected between 11 and 11.5 months of age.

ESM Table 2: Adipose tissue weights

	Male offspring				Female offspring			
	**Con** *n=12*	**Ob** *n=8*	**Ob-Met** *n=11*	p	**Con** *n=12*	**Ob** *n=12*	**Ob-Met** *n=11*	*p*
**Absolute weight (mg)**								
**BW (g)**	37.2 ± 1.0	41.8 ± 1.9	40.5 ± 2.1	ns	29.9 ± 0.9	31.0 ± 1.1	35.1 ± 1.4**,††	<0.001
**gWAT**	1771 [1206-2170]	2437** [2255-2920]	2288 [1569-2307]	<0.01	1203 ± 102	1504 ± 135	2053 ± 147***,††	<0.001
**iWAT**	539 ± 44	933 ± 80**	854 ± 99*	<0.01	466 ± 42	621 ± 51	809 ± 73***	<0.001
**rWAT**	409 ± 37	410 ± 32	379 ± 32	ns	244 ± 28	274 ± 21	341 ± 23*	<0.05
**sWAT**	782 ± 111	1264 ± 115	1239 ± 186	<0.05	795 ± 81	1083 ± 115	1632 ± 182***,††	<0.001
**VAT**	2603 ± 242	3834 ± 231*	3237 ± 301	<0.05	1913 ± 165	2376 ± 208	3203 ± 235***,††	<0.001
**BAT**	111 ± 8	158 ± 16	157 ± 21	0.058	74 ± 5	80 ± 6	114 ± 10**,†††	<0.001
**Relative to BW (%)**								
**gWAT**	4.47 ± 0.33	5.95 ± 0.20**	5.12 ± 0.20*	<0.01	3.96 ± 0.23	4.76 ± 0.29	5.78 ± 0.21***,††	<0.001
**iWAT**	1.46 ± 0.09	2.21 ± 0.14**	2.04 ± 0.16**	<0.01	1.53 ± 0.10	1.98 ± 0.12*	2.26 ± 0.11***	<0.001
**rWAT**	1.09 ± 0.08	0.99 ± 0.08	0.92 ± 0.05	ns	0.80 ± 0.07	0.86 ± 0.05	0.97 ± 0.04	ns
**sWAT**	2.04 ± 0.25	3.00 ± 0.19	2.90 ± 0.35	<0.05	2.61 ± 0.19	3.42 ± 0.26	4.53 ± 0.33***,††	<0.001
**VAT**	7.01 ± 0.52	9.15 ± 0.31**	8.22 ± 0.29	<0.01	6.29 ± 0.38	7.87 ± 0.33***	9.01 ± 0.32†	<0.001
**BAT**	111 ± 8	158 ± 16	157 ± 21	<0.05	235 [220-275]	240 [220-310]	300***,†† [280-370]	<0.01

All data reflects depot weights from 16-hour fasted animals at 12 months of age. **p*<0.05, ***p*<0.01, ****p*<0.001 vs Con offspring, †*p*<0.05, ††p<0.01, †††*p*<0.001 vs Ob offspring on one-way ANOVA with Tukey’s multiple comparison test or Kruskal-Wallis test for non-parametric data (absolute male gWAT and relative female BAT weight). Con = offspring of control-fed dams, Ob = offspring of obese dams, Ob-Met = offspring of obese metformin-treated dams. BAT = brown adipose tissue, gWAT = gonadal WAT, iWAT = intraperitoneal WAT, rWAT = retroperitoneal WAT, sWAT = inguinal subcutaneous WAT, VAT = visceral adipose tissue (combined gWAT, rWAT and sWAT), WAT = white adipose tissue (combined VAT + sWAT).

ESM Table 3: Age-related changes in epididymal adipocyte number

Male offspring

x 10^6 cells	Con	Ob	Ob-Met	*p*-value	Two-way ANOVA		
8 weeks	9.1 ± 0.2	11.8 ± 0.4***	13.3 ± 0.5***,†	<0.001	**Age**	**Group**	**Interaction**
12 months	13.9 ± 1.2§	18.2 ± 0.8*,§§	14.9 ± 1.4	0.0393	*p*<0.001	*p*=0.0015	*p*=0.045<br>Con§, Ob§

Female offspring

x 10^6 cells	Con	Ob	Ob-Met	*p*-value	Two-way ANOVA *p*-values		
8 weeks	9.5<br>[8.1–10.7]	10.1<br>[8.1–11.2]	10.8<br>[8.8–11.5]	ns	**Age**	**Group**	**Interaction**
12 months	11.5 ± 0.4	10.9 ± 0.6	13.2 ± 0.6†	0.0169	*p*<0.001	*p*=0.0227<br>Ob vs Ob-Met***	ns

$^{\S}p<0.01$, $^{\S\S}p<0.001$ for age-related change in cell number. $^{*}p<0.05$ vs Con, $^{***}p<0.001$ vs Con, $^{\dagger}p<0.05$ vs Ob offspring on one-way (left columns) or two-way ANOVA (right columns). Data from 8-week-old mice was previously published by Schoonejans *et al.* 2021 [10].

ESM Figure 1: Hepatic collagen content in male (a) and female (b) 12-month-old offspring assessed by picrosirius red staining.

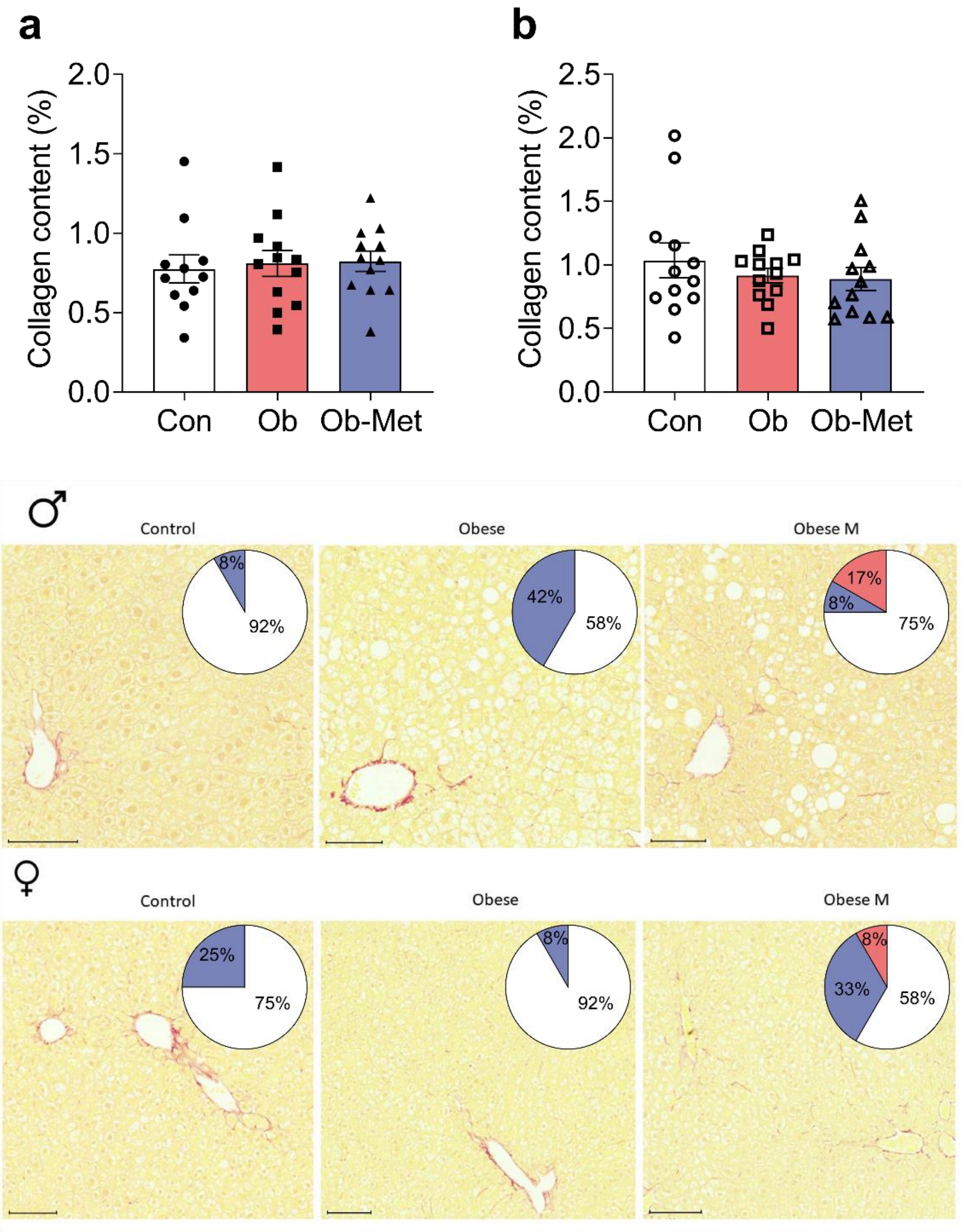

No significant differences were found. Pie charts refer to outcomes of pathological scoring for hepatic fibrosis: white = absent, blue = mild, light red = moderate. An outlier was excluded from (a) male Con (Grubb's method, outlier excluded value 2.30%). Con (white bars, circles) = offspring of control-fed dams, Ob (pink bars, squares) = offspring of obese dams, Ob-Met (blue bars, triangles) = offspring of obese metformin-treated dams. Closed symbols denote male offspring, open symbols denote female offspring. Scale bar = 100µm.
